# Effect of Missing Data Imputation on Deep Learning Prediction Performance for Vesicoureteral Reflux and Recurrent Urinary Tract Infection Clinical Study

**DOI:** 10.1155/2020/1895076

**Published:** 2020-07-15

**Authors:** Timur Köse, Su Özgür, Erdal Coşgun, Ahmet Keskinoğlu, Pembe Keskinoğlu

**Affiliations:** ^1^Ege University Faculty of Medicine, Department of Biostatistics and Medical Informatics, Turkey; ^2^Genomics Team, Microsoft Research, Redmond, WA, USA; ^3^Ege University Children's Hospital, Department of Pediatric Nephrology, Turkey; ^4^Dokuz Eylul University Faculty of Medicine, Department of Biostatistics and Medical Informatics, Turkey

## Abstract

Missing observations are always a challenging problem that we have to deal with in diseases that require follow-up. In hospital records for vesicoureteral reflux (VUR) and recurrent urinary tract infection (rUTI), the number of complete cases is very low on demographic and clinical characteristics, laboratory findings, and imaging data. On the other hand, deep learning (DL) approaches can be used for highly missing observation scenarios with its own missing ratio algorithm. In this study, the effects of multiple imputation techniques MICE and FAMD on the performance of DL in the differential diagnosis were compared. The data of a retrospective cross-sectional study including 611 pediatric patients were evaluated (425 with VUR, 186 with rUTI, 26.65% missing ratio) in this research. CNTK and R 3.6.3 have been used for evaluating different models for 34 features (physical, laboratory, and imaging findings). In the differential diagnosis of VUR and rUTI, the best performance was obtained by deep learning with MICE algorithm with its values, respectively, 64.05% accuracy, 64.59% sensitivity, and 62.62% specificity. FAMD algorithm performed with accuracy = 61.52, sensitivity = 60.20, and specificity was found out to be 61.00 with 3 principal components on missing imputation phase. DL-based approaches can evaluate datasets without doing preomit/impute missing values from datasets. Once DL method is used together with appropriate missing imputation techniques, it shows higher predictive performance.

## 1. Introduction

The unique guidelines have been developed for the differential diagnosis of diseases based on the literature and new research results. These guidelines for diseases are prepared by both evaluating multivariable mathematical model results and clinical experiences. In this study, the prediction performances of different statistical approaches for differential diagnosis (which is very difficult to predict) were compared in the presence of high incomplete data ratio on vesicoureteral reflux- (VUR-) recurrent urinary tract infection (rUTI) database.

### 1.1. The Importance of VUR/rUTI Differential Diagnosis

Vesicoureteral reflux is a congenital urinary tract anomaly of urine from the bladder to the kidney which is diagnosed mostly after an episode of recurrent urinary tract infection [1, 2]. VUR affects 1-2% of children [3] and depending on age is much higher among children with rUTI (15-70%) [4]. VUR should be considered in children who have urinary tract anomalies diagnosed by fetal ultrasonography and who have recurrent UTI. VUR was detected in 25-40% of pediatric patients with primary or recurrent UTIs [5]. When children with VUR have recurrent urinary tract infections, reflux nephropathy and renal dysfunction are more common [6]. About one-third of children with UTI are found to have VUR. rUTI is a finding of VUR and causes delay of VUR diagnosis due to differential diagnosis problem. This process can result in kidney damage. Therefore, it is important to make the diagnosis early.

Imaging methods are used for diagnosis in cases where VUR is suspected. Voiding cystourethrography (VCUG) is the gold standard radiographic exam to diagnose VUR [7]. Moreover, renal/bladder ultrasound (RBUS) which is a noninvasive procedure is generally used as an initial screening test to assess VUR. But diagnostic accuracy of RBUS is controversial because RBUS has been reported with low sensitivity and specificity for diagnosing VUR in children with UTI in some studies. Generally, the diagnostic value of ultrasound with clinical and laboratory findings is not evaluated in many studies [8–11].

The presence of renal scarring changes the management of VUR. Early diagnosis of VUR should be performed to prevent the development of renal scar, and prognosis of the patient should be well monitored. Additionally, the accuracy of early diagnosis and prognostic monitoring methods should be known.

Recently, some different analytical methods such as an artificial neural network (ANN) together are used to determine the accuracy of diagnostic tests and differential diagnoses with classical approaches. Classical approaches and some machine learning approaches lead to misinterpretations when incomplete data structures are encountered. Therefore, in cases where the differential diagnosis is very important and incomplete observations are inevitable (such as differential diagnosis of VUR and rUTI), it is important to increase the predictive power by using methods together such as deep learning with multiple imputation techniques [12].

### 1.2. Statistical Approaches for Differential Diagnosis

The discovery of information from big data has gained importance due to the development of computer technologies. Due to the size of data and the variety of this data, classical statistical approaches could not solve the problems of the researchers. Therefore, machine learning and deep learning methods have been popular in the medical field for researchers in recent years.

Computerized algorithms have been created to ingest rectangular datasets. In these datasets, rows represent observations and the columns represent variables. Matrices of these datasets contain elements whose values are real numbers. In many datasets, due to some reasons, some of the elements of the matrix are not observed. That leads to a dilemma for the analyst who is using techniques that require a full data matrix. An analyst must make a decision about the actual reason underlying unobserved variables. The easiest way to provide this condition is to delete these observations for analysts. It is almost impossible to obtain data without loss observation in real life. The use of case-wise deletion has led to large errors in the variance and covariance of the estimates. The use of mean imputation for missing values leads to large errors in variance estimates when variables have linear relationships. Conversely, iterative imputation provides the lowest errors and ANN and SVR are ranked the lowest in data error reported [13].

Generally, missing data creates various problems, especially during the data preprocessing phase. First of all, the absence of data reduces statistical power, which means the possibility of rejecting the null hypothesis when the test is false. Then, lost data can cause bias in estimating mass parameters. Third but not last, there is a risk that the sample will be less representative. Each of these problems will cause the validity of the data obtained in difficult conditions to be questioned. It will also cause the results of the research to be unreliable [14]. Many statistical approaches have been proposed to prevent missing data from causing such problems. The use of these approaches in classical statistical hypothesis testing is common. However, how deep observation analysis, which is one of the new generation machine learning methods, affects the predictive performance of the missing observation has not been investigated sufficiently. A comprehensive study will guide researchers, especially for real clinical trials.

Missing data has always been a tough challenge in clinical studies too. Decision-making processes based on accurate information are highly dependent on the completeness of data from the information source that can be obtained. However, real-world data tends to be incomplete, noisy, and inconsistent. In some cases, data could be lost, corrupted, or recorded incompletely, which affects the quality of the data negatively. Machine learning frameworks such as support vector machines (SVM), artificial neural networks (ANN), random forest (RF), and principal component analysis (PCA) cannot be used for decision-making and data analysis if the dataset is highly incomplete. Therefore, evaluating the data with the right methods to cope with the missing observations is very important in order to make correct inferences [15].

Medical records are major sources for epidemiological and clinical research. However, it is almost impossible to obtain datasets without missing values in the real clinical applications. Missing data presents major challenges to research by reducing viable sample size and introducing potential biases through patient/case selection or imputation [16, 17]. Also, evaluation of missing values in the dataset with appropriate methods is important for the reliability of the results of the research. Depending on various reasons, missing values occur in the datasets. These are defined as missing completely at random (MCAR), missing at random (MAR), and missing not at random (MNAR) [18].

To illustrate these three definitions, let us consider a dataset of patients collected in a hospital. When a physician decides not to measure the patients' body temperature because he/she can already see that the temperature is too high, then we have the MNAR scenario—the decision of not measuring the parameter depends on its actual value. On the other hand, if the temperature is systematically measured, but sometimes data registration process malfunctions (independently on the measured values), then we have the MCAR scenario. Finally, when the physician has a habit of not measuring the temperature of patients with high blood pressure (and blood pressure is always registered), then we have a MAR scenario [19].

Previously, when many missing data are observed in the data, the rate of missing observation is tried to be reduced by deleting the observation or by simple assignment techniques. As computational resources have increased, complex multiple imputation techniques have become applicable. Multiple imputation has a number of advantages over these other missing data approaches. Multivariate imputation by chained equations (MICE) algorithm is frequently used in hybrid missing datasets. High prediction performances are obtained by imputation of missing values in categorical and continuous data with the use of this method [20].

One of the other frequently used imputation techniques in mixed data is the factor analysis of mixed data (FAMD) algorithm. In this method, the number of components that may be suitable for the data is determined and the imputation of missing values is assigned [21].

Zhang conducted his study by using MIMIC-II database involving >30000 patients and he generated 150 patients with simulation. There were roughly 30% missing values in the lactate variable. The MICE technique was used for the simulated dataset [22]. Schmitt et al. compared six methods for missing data imputation in their study. Comparison was performed on four real datasets of various sizes (from 4 to 65 variables), under a missing completely at random (MCAR) assumption. Mean produced the largest number of hits with more than 21000 results, followed by MICE, SVD (singular value decomposition), and KNN (*k*-nearest neighbour) (17600, 14500, and 12700, respectively) [23].

Fisher's Iris data, Pima Indian data, Prostate cancer data, and wine datasets were used for comparison of imputation methods using mean, median, KNN, IRMI (Iterative Robust Model-based Imputation), FAMD, and HotDeck algorithms. FAMD had similar percentages of the observations correctly classified regardless of the amount of missingness in the data [24].

Leha et al. used to predict pulmonary hypertension based on a broader set of echocardiographic data with little reliance on estimated RAP (right atrial pressure) compared to an existing formula with noninferior performance via five (random forest of classification trees, random forest of regression trees, lasso penalized logistic regression, boosted classification trees, and support vector machines) machine learning algorithms [25]. Before applying the ML algorithms, missing values were imputed using the “iterative FAMD algorithm” and extract components were obtained explaining most of the variance [21]. ML algorithms provided high prediction accuracy with random forest of regression trees (AUC 0.87).

Apart from these studies, deep learning- (DL-) based approaches have become one of the most popular methods of recent years due to the increase in computer technologies and providing solutions to problems that researchers consider impossible in data. DL-based approaches can analyze the dataset without doing preomit/impute missing values from dataset [26, 27]. In this study, the effects of multiple imputation techniques MICE and FAMD on the performance of deep learning algorithm in the differential diagnosis for VUR and rUTI were compared.

## 2. Materials and Methods

### 2.1. Materials

In this retrospective cross-sectional study, 611 pediatric patients who had been admitted to Ege University, Faculty of Medicine Pediatric Nephrology, Outpatient Clinic and Tepecik Education and Research Hospital in Turkey, were included. Informative data about the patients were obtained from hospital records and patient files. The conversion of records into data was carried out by pediatric nephrologists in the research study team, and a database was created by the same team. Therefore, data collection and database have a consistent information [28]. The variables determined by the nephrologists for the study were presented in Table 1.

### 2.2. Methods

In this study, MICE and FAMD multiple assignment methods against deep learning algorithm estimation performance in missing data were evaluated for the differential diagnosis of VUR and rUTI, which are the most confused and difficult to distinguish in the clinic. Data store starts once patient's laboratory or USG test is performed only at the request of the clinician. In order to diagnose VUR or rUTI, the physician evaluates physical findings, imaging, and laboratory examinations of all patients. Therefore, missing values in the patient's files satisfy the MCAR or MAR preassumptions.

#### 2.2.1. MICE

Disregarding incompleteness or handling the data unsuitably may bias study results, decrease power and efficiency, and alter important risk/benefit relationships. Hence, classical techniques like single imputations are generally inappropriate due to the loss of precision and risk of bias [29]. Multiple imputations by multivariate imputation by chained equations method (“MICE”) are a powerful and statistically valid method for creating imputations in large datasets which include both categorical and continuous variables. Also, MICE is one of the most frequent methods used to replace missing data values in a dataset under certain assumptions about the data missingness mechanism [30, 31]. MICE has come out in the statistical literature as one principled method of addressing missing data, called “fully conditional specification” or “sequential regression multiple imputations.” MICE works under the assumption that given the variables used in the imputation procedure, the missing data are missing at random (MAR), which means that the probability that a value is missing depends only on observed values and not on unobserved values [30]. But implementing MICE when datasets are not appropriate for MAR or MCAR could result in biased estimates.

The steps of MICE process are as follows.

**Step 1 .**

A simple imputation such as “mean” is performed for each missing value in the dataset. These “mean” imputations can be thought of as “place holders.”

**Step 2 .**

The “place holder” mean imputations for one variable (“var”) are set back to missing.

**Step 3 .**

The observed values from the variable “var” in Step 2 are regressed on the other variables in the imputation model, and “var” is the dependent variable in a regression model. The remaining variables are independent variables in the regression model. These regression models work under the same assumptions that one would make when performing linear, logistic, or Poisson regression models outside of the context of imputing missing data.

**Step 4 .**

Then, missing values for “var” are replaced with predictions (imputations) from the regression model. When “var” is subsequently used as an independent variable in the regression models for other variables, both the observed and these predicted (imputed) values will be used.

**Step 5 .**

Steps 2–4 are then repeated for each variable that has missing data. The cycling through each of the variables constitutes a “cycle.” At the end of one cycle, all of the missing values have been replaced with predictions from regressions.

**Step 6 .**

Steps 2–4 are repeated for a number of cycles, with the imputations being updated at each cycle [32].

**Figure 1:**
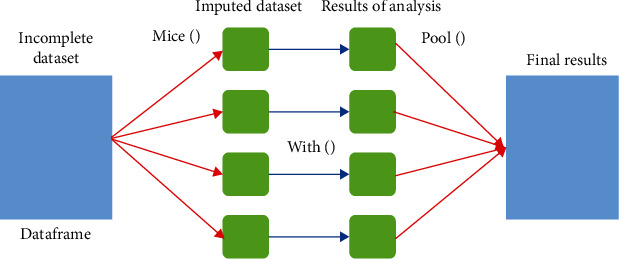
Multiple imputation via MICE package.

The MICE algorithm can impute mixes of continuous, binary, unordered categorical and ordered categorical data. MICE is very flexible in multiple imputation procedures, and it can be used in a broad range of settings. Because multiple imputations involve creating multiple predictions for each missing value, the analyses of multiply imputed data consider the uncertainty in the imputations and yield accurate standard errors [32]. On a simple level, if there is not much information in the observed data (used in the imputation model) regarding the missing values, the imputations will be very variable, leading to high standard errors in the analyses. In contrast, if the observed data are highly predictive of the missing values, the imputations will be more consistent across imputations, resulting in smaller, but still accurate, standard errors (Figure 1) [33].

#### 2.2.2. FAMD

Factor analysis of mixed data (FAMD) is a principal component method which balances the influence of all the variables that are continuous and categorical in the construction phase of the dimensions of variability. It can be identified as a harmonization of PCA (principal component analysis) and MCA (multiple correspondence analysis). The aim of using these methods is to find similarities between individuals, the relationships between variables (here continuous and categorical variables), and to link the study of the individuals with that of the variables. These methods reduce the dimensionality of the data and provide the subspace that best represents the dataset. Continuous variables of the dataset are scaled to unit variance, and the categorical variables are transformed into a disjunctive data table and then scaled using the specific scaling of MCA. This procedure provides balancing of the influence of both continuous and categorical variables in the analysis. FAMD method allows one to study the similarities between individuals considering different types of variables and to study the relationships between all the variables. It also ensures graphical outputs, representation of the individuals, the correlation circle for the continuous variables and representations of the categories of the categorical variables, and also specific graphs to visualize the associations between both types of variables [21].

The steps of the FAMD algorithm are as follows.

We represent using *I* as the number of individuals, *N*_1_ the number of continuous variables, *N*_2_ the number of categorical variables, and *N* = *N*_1_ + *N*_2_ the total number of variables.

**Step 7 .**

Coding the categorical variables using the indicator matrix of dummy variables. In *X*_*IxJ*_ matrix, continuous variables are shown with (*x*_*j*_)_1≤*j*≤*N*_1__ and dummy variables are shown with (*x*_*j*_)_*N*_1_+1≤*j*≤*J*_. The total number of column is *J* = *N*_1_ + ∑_*n*=*N*_1_+1_^*N*^*qn*, where *q*_*n*_ is the number of categories of the variable *n*.

**Step 8 .**

This step is defined as the weighting step and each continuous variable (*x*_*j*_) is divided by its standard deviation (*s*_*j*_). Thus, standardized values are obtained. Also, each dummy variable is divided by $\sqrt{p_{j}}$, where *p*_*j*_ denotes the proportion of individuals that take the category *j* (*j* = *N*_1_ + 1, ⋯, *N*).

**Step 9 .**

FAMD consists in performing a PCA on the weighted matrix **XD**_∑_^−1/2^. In this weighted matrix, *D*_∑_ is defined as *s*_*x*1_^2^, ⋯, *s*_*xN*_1__^2^, *p*_*N*_1_+1_, ⋯, *p*_*j*_, ⋯, *p*_*J*_. **XD**_∑_^−1/2^ − **G**, singular value decomposition (SVD) of the matrix with **G**_*IxJ*_ the matrix with each row equals to the vector of the means of each column of **XD**_∑_^−1/2^. The first *S* dimensions of variability are preserved as in any principal component methods.

The specific weighting implies that the distances between two individuals *i* and *i*′ in the initial space are as follows:

(1) $$\begin{array}{c} d^{2}\left(i,i^{\prime }\right)=\overset{N_{1}}{\underset{n=1}{\sum }}\frac{{\left(x_{\text{in}-x_{i^{\prime }n}}\right)}^{2}}{s_{xn}^{2}}+\overset{J}{\underset{j=N_{1}+1}{\sum }}\frac{1}{p_{j}}\;{\left(x_{ij}-x_{i^{\prime }j}\right)}^{2}. \end{array}$$

**Figure 2:**
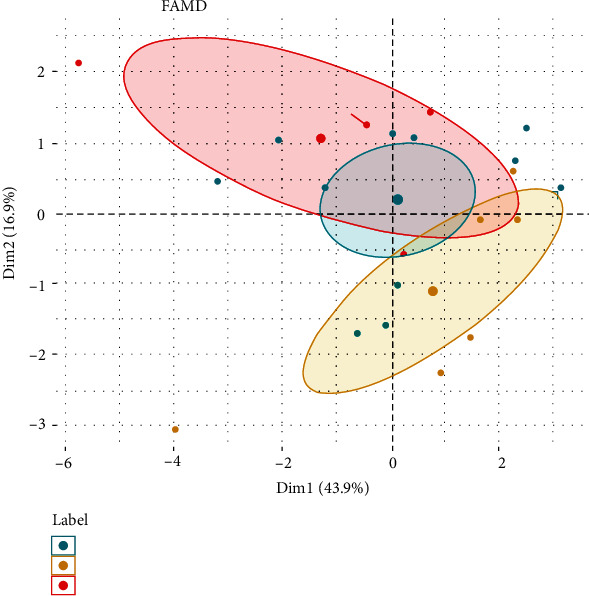
FAMD factor map. Analyzing mixed data [36].

Weighting by 1/*s*_*x*_*n*__^2^ keeps that units of continuous variables do not influence the (square) distance between individuals (Figure 2) [34, 35].

#### 2.2.3. Deep Learning

Having missing values is a much known problem in statistical analysis. Most of the statistical methods cannot be directly applied on an incomplete dataset due to their mathematical assumptions. Deep learning-based approaches can evaluate the datasets without doing preomit/impute missing value from dataset [37].

Deep learning is an artificial intelligence function that imitates the workings of the human brain for processing data and creating patterns for use of decision-making. Deep learning is a subset of machine learning in artificial intelligence (AI) that has a network architecture. These networks are capable of learning data with unsupervised approach from unstructured or unlabeled format. During the training process, algorithms use unknown elements in the input distribution to extract features, group objects, and discover useful data patterns. Much like training machines for self-learning, this occurs at multiple levels, using the algorithms to build the models.

**Figure 3:**
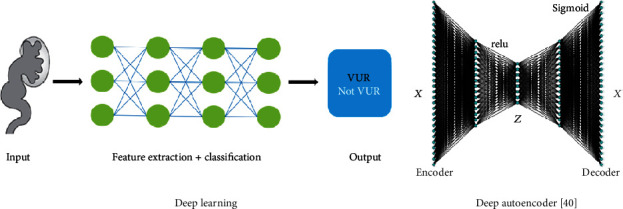
Deep learning. Deep autoencoder [40].

Deep learning requires the use of many hidden neurons and layers with new training models as an architectural advantage (Figure 3). The use of a large number of neurons allows a comprehensive representation of the available raw data. Adding more hidden layers to the neural network allows hidden layers to capture nonlinear relationships. When the neural network is optimally weighted, there is effective high-level representations of obtained raw data or images [38, 39].

Deep learning models have important hyperparameters such as learning rate, batch size, and epoch. Finding the best configuration of these hyperparameters in a high dimensional space directly affects the performance of the estimations.

**Figure 4:**
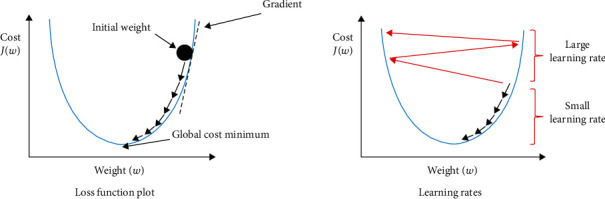
Hyperparameters of deep learning algorithm [41].

*(1) Gradient Descent*. This optimization technique is widely used in the training of machine learning algorithms. The main purpose of training machine learning algorithms is to adjust the weights “*w*” of variables (inputs) to minimize loss or cost. This cost “*J* (*w*)” represents the performance of the model and optimal parameters may obtain by minimizing the cost function (Figure 4).

*(2) Learning Rate*. Gradient descent algorithms multiply the gradient (slope) by a scalar known as the learning rate to determine the next point, and weights are updated during training according to the learning rate.

*(3) Batch Size and Epoch*. One “epoch” is completed when an entire dataset is passed forward and backward through the neural network exactly one time.

The “batch” size is defined as a hyperparameter of gradient descent that controls the number of training samples to work through before the model's internal parameters are updated. The number of iterations is equivalent to the number of batches needed to complete one epoch. When a dataset includes 500 cases split into minibatches of 50 cases, it will take 10 iterations to complete a single epoch [41].

#### 2.2.4. CNTK

Depending on the increasing data sources and the size of the data in the medical field, classical statistical approaches were insufficient in the analysis of large and complex structured data. The need for computer-aided systems that can evaluate clinical, laboratory, imaging, and genetic data together for the diagnosis and prognosis of diseases as well as analyze complex databases for planning healthcare services has increased. CTNK (Microsoft Cognitive Toolkit) provides that it enables the researcher to estimate the diagnosis and prognosis very quickly in databases with large and complex relationships. CNTK is a deep learning framework developed by Microsoft Research. CNTK describes neural networks as a series of computational steps via a directed graph [42]. Several industry-leading low-level deep learning libraries (Microsoft Cognitive Toolkit (CNTK), Tensorflow, Caffe, Torch, and MXNet) are used to support GPU acceleration. Besides the support of languages such as CNTK, Python, and C ++, it is highly optimized with efficient resource consumption. We have used CNTK for getting the advantages of fully cloud-capable environment. In the training phase of the DL model, the CNTK GPU library was used with 1000 layers, 10 minibatches, 100 epochs, and 0.0001 learning rate.

#### 2.2.5. Performance Measures

In this study, the performance of algorithms was evaluated with sensitivity, specificity, and accuracy. Definitions of the concepts are explained by confusion matrix Table 2.

When a disease is proven present in a patient, the given diagnostic test also indicates the presence of disease. In this case, the result of the diagnostic test is considered true positive (TP). Also, if a disease is proven absent in a patient, the diagnostic test suggests the disease is absent as well, the test result is true negative (TN). Unfortunately, no medical test is perfect. When the diagnostic test indicates positive the presence of disease in a person who is healthy, the test result is false positive (FP). In addition, when the result of the diagnosis test suggests that the disease is absent for a patient with disease for sure, the test result is false negative (FN).

Sensitivity, specificity, and accuracy are identified in terms of TP, TN, FN, and FP.

- Sensitivity = TP/(TP + FN) (Number of true positive assessment)/(Number of all positive assessment)
- Specificity = TN/(TN + FP) (Number of true negative assessment)/(Number of all negative assessment)
- Accuracy = (TN + TP)/(TN + TP + FN + FP) (Number of correct assessments)/(Number of all assessments) [43, 44]

#### 2.2.6. Statistical Analysis

Statistical analyses were performed using CNTK and R 3.6.3.10-fold cross-validation technique was used to evaluate predictive models by partitioning the original sample into a training set to train the model and a test set to evaluate it. In 10-fold cross-validation, the original sample was randomly partitioned into 10 equal-sized subsamples. Of the 10 subsamples, two subsamples were retained as the validation data for testing the model, and the remaining 8 subsamples were used as training data.

Parameter optimization is performed for FAMD-multiple missing imputation technique for the number of components. Optimization analysis showed that the number of components should be maximum 10. We have reported the results for different number of components: 2, 3, 6, and 10 for better representation of importance of optimization.

The values of sensitivity, specificity, and accuracy which are obtained from DL, MICE, and FAMD with DL were compared in Table 3.

#### 2.2.7. Ethics

Ethical approval for this study was obtained from the Board of Ethical Committee of Ege University (Protocol No. 13-6.1/56; the date of approval: 29.07.2013). Patients have given informed consent for participation in the study.

## 3. Results

**Figure 5:**
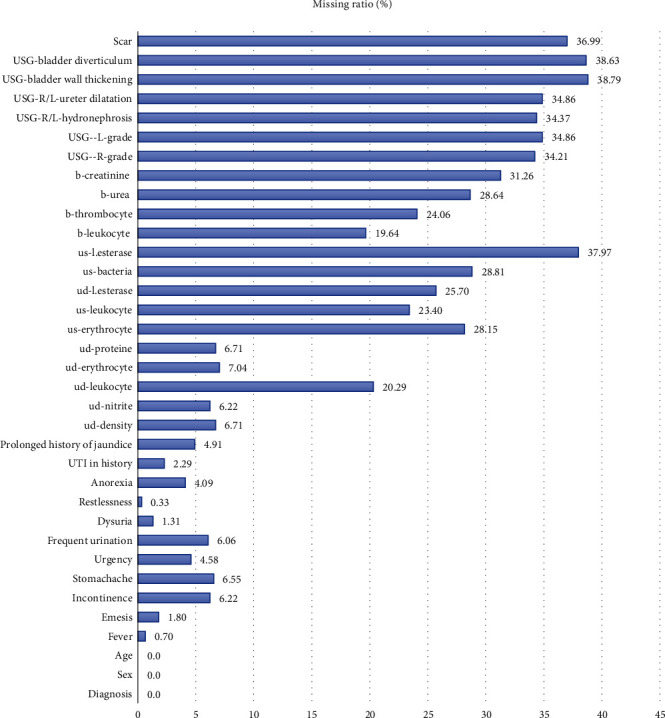
Missing ratio of variables in original (not imputed) dataset. UTI: urinary tract infection; ud: urine dipstick; us: urine sediment; USG: ultrasonography; b: blood; R: right; L: left; AP: anterior-posterior; u-le: urine-leukocyte esterase.

In this study, the data of 425 (69.6%) VUR and 186 (30.4%) rUTI children from the patient records were evaluated retrospectively. Performance without preimputation and imputed with MICE and FAMD methods was evaluated for the differential diagnosis of VUR/rUTI. The missing ratio of variables in the dataset is presented in Figure 5. As we reported, data has many variables which have more than 30% missing observation. We observed high missing ratio level for most of the USG records (Figure 5).

Correlations between continuous variables were presented in Table 4. Low grade correlations were found between measurements.

Descriptive statistics of original (not preimputed) and imputed ((MICE and FAMD) datasets were presented at Table 5. FAMD algorithm was applied to the dataset for 2, 3, 4, and 10 components. CNTK was used for evaluating different models for 34 features (physical findings, laboratory, and imaging findings). Deep neural network implementation was finalized with 128 hidden layers and L1 and L2 = 0.001 selection. Epoch value is 5 and numbers of iterations are 800 for final training model. Testing/training sample ratio is 20/80 for the entire analysis. 10-fold cross validation results were presented in Table 3. Accuracy, sensitivity, and specificity results of deep learning were, respectively, 57.65, 58.09, and 57.32. FAMD algorithm's best performance results were found, respectively, 61.52, 60.20, and 61.00. In the differential diagnosis of VUR and rUTI, the best performance was obtained with MICE algorithm; its values were, respectively, 64.05 accuracy, 64.59 sensitivity, and 62.62 specificity (Table 3).

## 4. Discussion

In this study, hospital records of children with VUR and rUTI, who were followed up in tertiary hospitals, were used. There were missing variables of up to 38% in different variables in the dataset due to reasons such as the lack of information in the retrospective hardcopy records of patients with long follow-up and the lack of examinations performed prior to admission to these centers.

Healthcare records contain a lot of missing values which imposes difficulties for researchers who plan to model these datasets. Clinical datasets, especially for laboratory measurements, and imaging records often contain missing values [45]. These shortcomings bring difficulties to capture the patterns in clinical datasets.

Machine learning frameworks such as support vector machines, artificial neural networks, random forest, and principal component analysis cannot be directly used for decision-making/data analysis if the dataset is incomplete. Therefore, we must preprocess the data before modeling phase.

In some cases, instead of dealing with missing values, researchers consider removing missing observations from the data. Removing missing observations may end with loss of information and biased assessments in results. Another approach is to use appropriate classical or multiple imputation techniques for missing observations.

A general assumption that is often made when using these imputation methods is that the data is *missing completely at random* or *missing at random.* Similar approaches were used in a simulation study on substance abuse and a study examining electronic health records [46, 47]. It is important to be able to evaluate data using techniques that allow or handle missing observations.

In missing data analysis literature, researchers performed the similar methodologies that we have used. Reported results are very similar across the different domains, disease OR data collection methods. Researchers usually impute their data with MICE and FAMD techniques, which are frequently used methods for hybrid (mixed) data [20, 21, 48]. Multiple imputations for missing data make it possible for the researcher to obtain approximately unbiased estimates of all the parameters from the random error. Multiple imputations for missing data allow the researcher to obtain good estimates of the standard errors.

Zhang has examined the relationship between lactate level and mortality using the MIMIC dataset; he set the missing value ratio to 30%. He reported that MICE had the highest approximation to the real/expected distribution of data. Also, he advised that using MICE imputation varieties of expressions can be executed including univariate analysis and multivariable regression models [22]. We have concluded the similar highest accuracy with MICE in our study. This similarity should be considered for the upcoming clinical database/trail outcomes. We recommended performing MICE as a baseline missing imputation approach for every study. On the other hand, Schmitt et al. tested 4 different missing imputations at various size datasets: small (breast cancer_1_-breast cancer_2_) and large (E-coli, Iris) and MICE brought the second-highest git. The dataset that we analyzed did not allow us to compare the results because of the high missing observation for SVD and KNN.

If we compare our studies' results with Hunt's study (containing prostate cancer, wine, Fisher's Iris, and Pima Indian dataset), FAMD with ‘ncomp = 3's prediction result is very close with MICE performance on DL training [24].

This shows us to validate the FAMD performance usually close to MICE on highly incomplete clinical data. Major assumption of this conclusion is “ncomp” optimization and selection. This phase is very critical for reaching out the highest prediction accuracy.

One of the methods that has become immensely popular in recent years is deep learning. With DL models, researchers can use all existing data without adding synthetic reputations unlike machine learning algorithms. On the other hand, it is possible to make more accurate estimates and obtaining results with fast, reliable, and repeatable analysis [49]. When using DL, the use of a large number of neurons ensures extensive representation of the available raw data, even if there are missing observations in the dataset. When the studies in the literature are evaluated, although deep learning techniques are frequently used in recent studies, there is no study in which missing imputation techniques and their performance are evaluated together. When multiple imputations were made, a 5% performance increase was observed compared to the estimation made only with the deep learning algorithm. In cases where the differential diagnosis is very difficult like VUR/rUTI, this increase is very important clinically.

During deep learning analysis, we have tried to keep the number of layers as high as possible. The main purpose of this choice was to create the network on GPU compute instance, with the highest efficiency. It is used at the highest level since it is not intended to make comparisons for the number of layers. Likewise, the number of minibatches was kept at the level of 10, which is at the level allowed by our computed environment. The other parameter, “epoch,” once we reviewed the studies in the relevant literature, the number of epoch was observed between 10 and 100. In this study, we used the highest possible value of 100, which is within our technical capabilities. Although it is generally accepted that the accuracy value will increase as epoch value increases, we would like to state that this value is used in the highest possible scenario for our study. When artificial neural network studies are observed, we aimed to get the lowest value used for the learning rate. Therefore, the value of 0.0001, which will affect the estimation results, was used in the test dataset. As it is well known, high selection of the learning rate may cause overfitting; therefore, this preference has been made by our research group. On the other hand, we have reported the optimum values that we used for all these parameters that can set a template for many researchers rather than comparing all the parameters with different levels. These values may change in other studies; therefore, we have tried to use the optimal values for our dataset. We have observed that there are optimal values that can be applied for such clinical dataset like ours. Unfortunately, optimal values have not been reported in similar clinical trials on literature. We hope that this study's parameter selection on deep learning models will be used as an example for other clinical studies. As in all other machine learning studies, like this study, it is foreseen to have similar missing value rates in order to generalize the prediction results. However, the deep learning method does not require deep data preprocessing operations. The major scientific value of the study is showing the usability of DLs with the fastest and accurate way. We hope that clinical studies will be analyzed with different deep learning parameter ranges in future studies.

## 5. Conclusions

It has been known for many years that the rate of missing observation is a major problem in real clinical trials. The techniques applied in most of the studies on this research area are to exclude one or more variables from analysis or to apply some statistical transformations to existing data. Thanks to the different method we applied in our study, researchers may consider the benefits of GPU-based missing data imputation methods. As it is known, clinical data have different characteristics for each disease, every new drug, and every new treatment method. Therefore, we have limited our conclusions and results just for the application data as we used on the study title. That was one of the limitation aspects of our study and we focused to highlight this very clearly. We hope that we will have an opportunity to apply this approach on different clinical datasets: medical image genomics or electronic medical records.

In conclusion, unlike machine learning techniques, deep learning allows estimation with incomplete datasets. It is suggested that the deep learning algorithm should be used together with appropriate imputation techniques for hybrid-type datasets for achieving the highest accuracy rates.

## Figures and Tables

**Table 1:** The variables used in deep learning and multiple imputation techniques.

Clinical variables	Laboratory variables	USG variables
Diagnosis_(VUR/rUTI)_	ud-density_(c)_	USG-R-grade _(ordinal:0,1,2)_
Sex_(cat: male/female)_	b-leukocyte_(c)_	USG-L-grade_(ordinal: 0,1,2)_
Age_(c)_	ud-nitrite_(cat:Y/N)_	USG-R/L hydronephrosis _(cat: Y/N)_
Fever_(cat: Y/N)_	ud-l.esterase_(cat: Y/N)_	USG-bladder wall thickening_(cat: Y/N)_
Emesis_(catty/N)_	ud-protein_(cat: Y/N)_	USG-bladder diverticulum_(cat: Y/N)_
Incontinence_(cat: Y/N)_	us-erythrocyte_(cat: Y/N)_	USG-ureter dilatation R/L _(cat: Y/N)_
Stomachache_(cat: Y/N)_	us-leukocyte_(cat: Y/N)_	
Urgency_(cat: Y/N)_	ud-leukocyte_(cat: Y/N)_	
Frequent urination_(cat: Y/N)_	us-bacteria _(cat: Y/N)_	
Dysuria_(cat: Y/N)_	ud-erythrocyte_(cat)_	
Restlessness_(cat: Y/N)_	b-thrombocyte_(c)_	
Anorexia_(cat: Y/N)_	b-urea_(c)_	
UTI in history_(cat: Y/N)_	b-creatinine_(c)_	
Prolonged neonatal jaundice_(cat: Y/N)_		
Scar_(cat: Y/N)_		

All categorical variables are defined as binary (cat: Y/N, yes/no, and sex, cat: male/female). c: continuous variable; cat: categorical variable; rUTI: recurrent urinary tract infection; ud: urine dipstick; us: urine sediment; USG: ultrasonography; b: blood; R: right; L: left; u-le: urine-leukocyte esterase.

**Table 2:** Confusion matrix.

Diagnostic test	Gold standard			
	Positive	Negative	Row total	
Positive	TP	FP	TP+FP (total number of subjects with positive test)	Positive predictive value TP/(TP+FP)
Negative	FN	TN	FN+TN (total number of subjects with negative test)	Negative predictive value TN/(FN+TN)
Column total	TP+FN (total number of subjects with given condition)	FP+TN (total number of subjects without given condition)	N=TP+TN+FP+FN (total number of subjects in the study)	
	Sensitivity TP/(TP+FN)	Specificity TN/(FP+TN)		

TP: true positive; TN: true negative; FN: false negative; FP: false positive.

**Table 3:** 10-fold cross-validation results on CNTK.

	Accuracy	SD	Sensitivity	SD	Specificity	SD
Deep learning-original dataset	**57.65**	**4.18**	**58.09**	**4.09**	**57.32**	**7.32**
FAMD-ncomp = 2	58.55	3.58	58.99	3.99	59.52	5.62
FAMD-ncomp = 3	**61.52**	**1.08**	**60.20**	**0.99**	**61.00**	**1.32**
FAMD-ncomp = 6	58.85	0.10	55.89	0.16	65.92	0.26
FAMD-ncomp = 10	57.5	0.14	54.4	0.20	63.90	0.29
MICE	**64.05**	**4.38**	**64.59**	**0.09**	**62.62**	**6.12**

ncomp: number of component; SD: standard deviation.

**Table 4:** Correlations between continuous variables.

	Age	b-leukocyte	b-thrombocyte	b-urea	b-creatinine	ud-density
Age	1					
b-leukocyte	-0.176985	1				
b-thrombocyte	-0.154943	0.086716	1			
b-urea	0.303343	-0.067059	-0.035233	1		
b-creatinine	-0.001593	-0.039199	-0.033116	0.050520	1	
ud-density	0.278367	-0.075667	-0.158854	0.195563	0.064533	1

b: blood; ud: urine dipstick.

**Table 5:** Descriptive statistics of deep learning and multiple imputation techniques for the dataset.

Variables		Deep learning (original dataset)	MICE	FAMD (ncomp = 2)	FAMD (ncomp = 3)	FAMD (ncomp = 6)	FAMD (ncomp = 10)
Age	$\tilde{X}$ [Min-Max]	16 [0-196]	16 [0-196]	16 [0-196]	16 [0-196]	16 [0-196]	16 [0-196]
	$\bar{X}$ (1^st^-3^r^d quartiles)	36.6 (4.0-60.0)	36.6 (4.0-60.0)	36.6 (4.0-60.0)	36.6 (4.0-60.0)	36.6 (4.0-60.0)	36.6 (4.0-60.0)
b-leukocyte	$\tilde{X}$ [Min-Max]	10000 [950-110000]	10100 [271.5-110000]	10465 [950-110000]	10400 [950-110000]	10400 [950-110000]	10160 [950-110000]
	$\bar{X}$ (1^st^-3^r^d quartiles))	12205.4 (7500-10000)	12061.6 (7500-13651.1)	12175 (8100-13300)	12117 (8010-13021)	12155 (8000-13211)	12040 (7725-13100)
b-thrombocyte	$\tilde{X}$ [Min-Max]	341000 [17200-849000]	345000 [17200-849000]	345000 [17200-849000]	345000 [17200-849000]	346000 [17200-849000]	345100 [17200-849000]
	$\bar{X}$ (1^st^-3^rd^ quartiles)	358345.9 (17200-849000)	357035 (277274-849000)	358355 (296000-849000)	358223 (296000-396000)	358412 (296000-397087)	356165 (288000-400352)
b-urea	$\tilde{X}$ [Min-Max]	19.0 [0.40-112.0]	20.0 [0.4-112.0]	19.4 [0.4-112.0]	19.0 [0.4-112.0]	19.0 [0.4-112.0]	19.0 [0.4-112.0]
	$\bar{X}$ (1^st^-3^r^d quartiles)	20.3 (13.0-25.0)	20.4 (14.2-25.0)	20.1 (15.0-24.0)	20.1 (15.0-24.0)	20.2 (15.0-24.0)	20.2 (14.0-24.3)
b-creatinine	$\tilde{X}$ [Min-Max]	0.5 [0.10-143.9]	0.50 [0.02-143.0]	0.50 [0.1-143.0]	0.50 [0.1-143.0]	0.50 [0.014-143.0]	0.5 [0.013-143.0]
	$\bar{X}$ (1^st^-3^r^d quartiles)	1.24 (0.10-143.0)	2.16 (0.40-0.90)	1.27 (0.40- 0.98)	1.29 (0.40-1.00)	1.50 (0.40-0.90)	2.01 (0.40-1.46)
ud-density	$\tilde{X}$ [Min-Max]	1013.0 [1000-1035]	1013 [1000-1035]	1013 [1000-1035]	1013 [1000-1035]	1013 [1000-1035]	1013 [1000-1035]
	$\bar{X}$ (1^st^-3^r^d quartiles)	1013.77 (1006.0-1020.0)	1014 (1006-1020)	1013 (1006-1020)	1014 (1006-1020)	1014 (1006-1020)	1014 (1006-1020)
Stomachache	No	280	283	288	286	284	284
	Yes	96	98	96	96	97	99
	Not assessed	195	230	227	229	230	228
Urgency	No	307	313	322	321	317	316
	Yes	98	100	98	98	99	316
	Not assessed	178	198	191	192	195	195
Frequent urination	No	250	255	259	258	258	256
	Yes	94	99	95	96	95	98
	Not assessed	230	257	257	257	258	257
Dysuria	No	264	268	267	268	268	267
	Yes	92	92	92	92	92	92
	Not assessed	247	251	252	251	251	252
Restlessness	No	114	115	114	114	114	115
	Yes	476	477	478	478	478	477
	Not assessed	19	19	19	19	19	19
Anorexia	No	461	474	486	485	486	479
	Yes	72	79	72	72	72	77
	Not assessed	53	58	53	54	53	55
UTI history	No	136	141	136	137	136	141
	Yes	470	470	475	474	475	470
Prolonged history of jaundice	No	537	565	566	566	566	566
	Yes	44	46	46	46	46	46
Fever	No	364	368	368	368	368	367
	Subfebrile	64	64	64	64	64	64
	Febrile	179	179	179	179	179	180
Emesis	No	503	511	514	514	514	511
	Yes	97	100	97	97	97	100
Incontinence	No	262	273	278	275	274	273
	Yes	73	78	74	76	76	77
	Not assessed	238	260	259	260	261	261
ud-nitrite	No	511	547	549	549	549	547
	Yes	62	64	62	62	62	64
ud-protein	No	509	546	550	550	550	547
	Yes	61	65	61	61	61	64
us-erythrocyte	No	428	598	494	600	600	598
	Yes	11	13	117	11	11	13
us-leukocyte	No	386	502	521	524	524	509
	Yes	82	109	90	87	87	102
ud-leukocyte	No	342	444	483	476	474	450
	Yes	112	167	128	135	137	161
us-bacteria	No	378	536	551	552	539	538
	Yes	57	75	60	59	72	73
ud-erythrocyte	No	424	539	548	548	547	533
	Yes	63	72	63	63	64	78
ud-l.esterase	No	367	596	521	599	599	599
	Yes	12	15	90	12	12	12
USG-R-grade	Grade 0	384	592	593	593	593	592
	Grade 1	14	15	14	14	14	15
	Grade 2	3	3	3	3	3	3
	Grade 3	1	1	1	1	1	1
USG-L-grade	Grade 0	389	602	602	602	602	601
	Grade 1	5	5	5	5	5	6
	Grade 2	2	2	2	2	2	2
	Grade 3	1	1	1	1	1	1
	Grade 4	1	1	1	1	1	1
USG-R/L-hydronephrosis	Normal	340	546	546	548	546	545
	Mild	36	39	36	36	36	39
	Heavy	25	26	29	27	29	27
USG-R/L-ureter dilatation	No	365	576	578	577	576	576
	Yes	33	35	33	34	35	35
USG-bladder wall thickening	No	353	581	590	590	590	587
	Yes	21	30	21	21	21	24
USG-bladder diverticulum	No	365	600	601	601	601	599
	Yes	10	11	10	10	10	12
Scar	Normal	286	487	506	507	506	495
	Yes	99	124	105	104	105	116

UTI: urinary tract infection; ud: urine dipstick; us: urine sediment; USG: ultrasonography; b: blood; R: right; L: left; AP: anterior-posterior; u-le: urine-leukocyte esterase; $\bar{X}$: mean; $\tilde{X}$: median; Min: minimum; Max: maximum.

## Data Availability

Sharing this data with researchers is only possible with the approval of the ethics committee because this dataset is real clinical data. For this reason, in case the researchers who are interested in the data contact the corresponding author, the dataset will be shared in accordance with the data privacy rules. It is appropriate to present the text as stated in the article.
